# Prevalence and predictors of complementary and alternative medicine use among people with coronary heart disease or at risk for this in the sixth Tromsø study: a comparative analysis using protection motivation theory

**DOI:** 10.1186/s12906-017-1817-x

**Published:** 2017-06-19

**Authors:** Agnete E. Kristoffersen, Fuschia M. Sirois, Trine Stub, Anne Helen Hansen

**Affiliations:** 1 The National Research Center in Complementary and Alternative Medicine (NAFKAM), Faculty of Health Sciences, Department of Community Medicine, UiT The Arctic University of Norway, Tromsø, Norway; 2 Department of Psychology, University of Sheffield, Sheffield, UK; 3 University Hospital of North Norway and Faculty of Health Sciences, Department of Community Medicine, UiT The Arctic University of Norway, Tromsø, Norway

**Keywords:** Coronary heart disease, Cardiovascular disease, Health care utilization, Complementary and alternative medicine, CAM, Protection motivation theory, PMT, Norway

## Abstract

**Background:**

Engagement in healthy lifestyle behaviors, such as healthy diet and regular physical activity, are known to reduce the risk of developing coronary heart disease (CHD). Complementary and alternative medicine (CAM) is known to be associated with having a healthy lifestyle. The primary aim of this study was to examine the prevalence and predictors of CAM use in CHD patients, and in those without CHD but at risk for developing CHD, using Protection Motivation Theory (PMT) as a guiding conceptual framework.

**Method:**

Questionnaire data were collected from 12,981 adult participants in the cross-sectional sixth Tromsø Study (2007–8). Eligible for analyses were 11,103 participants who reported whether they had used CAM or not. Of those, 830 participants reported to have or have had CHD (*CHD group*), 4830 reported to have parents, children or siblings with CHD (*no CHD but family risk*), while 5443 reported *no CHD nor family risk* of CHD*.* We first compared the patterns of CAM use in each group, and then examined the PMT predictors of CAM use. Health vulnerability from the threat appraisal process of PMT was assessed by self-rated health and expectations for future health. Response efficacy from the coping appraisal process of PMT was assessed as preventive health beliefs and health behavior frequency.

**Results:**

Use of CAM was most commonly seen in people with no CHD themselves, but family risk of developing CHD (35.8%), compared to people already diagnosed with CHD (30.2%) and people with no CHD nor family risk (32.1%). All four of the PMT factors; self-rated health, expectations for future health, preventive health beliefs, and the health behavior index – were predictors for CAM use in the *no CHD but family risk group*.

**Conclusion:**

These findings suggest that people use CAM in response to a perceived risk of developing CHD, and to prevent disease and to maintain health.

## Background

Cardiovascular disease is the most common cause of death in Norway [1] and Europe [2]. However, mortality rates have decreased significantly over the past years both for coronary heart disease (CHD) and stroke [2], partly because survival after acute myocardial infarction has improved substantially [3]. Risk factors for the development of CHD include non-modifiable factors such as family history of CHD, and modifiable factors including health behaviors and stress [4]. Engagement in healthy lifestyle behaviors, such as healthy diet and regular physical activity, are known to reduce the risk of developing CHD [5–7]. Primary prevention of CHD involves encouraging individuals at risk to make healthy lifestyle changes to reduce the risk.

Increasingly, individuals interested in improving their health and making healthy lifestyle changes are turning to complementary and alternative medicine (CAM) as a self-care and health-care option [8, 9]. CAM use is associated with other healthy lifestyle behaviors such as diet and physical activity in national surveys from the U.S. [10, 11], Canada [12], Germany [13] and Australia [14]. Taken together with qualitative and quantitative research indicating that CAM providers help promote healthy lifestyles in their clients [15], this evidence indicates that individuals may apply CAM modalities as a means to achieve a healthy lifestyle, maintain health, and to reduce the chances of developing disease. Indeed maintaining a healthy lifestyle is generally accepted as important for reducing risk of developing diseases such as CHD, especially among those with increased risk due to non-modifiable factors [16]. Despite the known links between CAM use and engaging in healthy behaviors and the role of healthy behaviors in reducing modifiable CHD risk [4], little is known about the patterns and reasons for CAM use among those at risk for developing CHD.

Protection Motivation Theory (PMT) is a social cognitive model for predicting health behavior that may be useful for better understanding CAM use as a health protective behavior among those at risk for CHD [17–19]. This theory provides a framework for understanding motivations to engage in protective behaviors in response to health threats. Specifically, the PMT posits that a protection motivation results from two appraisal processes, a *threat appraisal*, followed by a *coping appraisal*. The threat appraisal involves assessing the perceived *severity* of the threat, as well as the probability of *being vulnerable* to the threat. This appraisal is based on *perceptions* of vulnerability, which may or may not rely on objective indicators of vulnerability for a particular health threat. Perceiving that one is at risk, for example by having non-modifiable risk factors, is enough to activate perceptions of vulnerability and corresponding health protective behaviors such as CAM use. Once a threat is perceived as being severe and involving personal vulnerability, a coping appraisal process is initiated to deal with the threat. The coping appraisal includes assessing the efficacy of the health behavior for dealing with the threat (*response efficacy*), as well as the individual’s self-efficacy or confidence for being able to engage in the behavior.

PMT is most commonly used for assessing how people respond to health risk messages, such as those in the media or delivered by a health-care professional about needed changes in health behavior to reduce risk of disease [20, 21]. However, PMT is also used for understanding a more general response to health threats including knowledge about one’s own risk for developing disease, based on risk factors such as family history of CHD, and the health protective behaviors that my reduce this risk [19].

Research describing prevalence and pattern of use of CAM in patients with CHD is sparse [22]. The few existing studies is in highly selected population subgroups, with substantial differences in proportion of CAM users ranging from 4%–85% [23–27]. Similar to patients with other chronic diseases, CHD patients are likely to use CAM to manage their condition, increase quality of life and prevent recurrence of their disease [28]. CAM use for general health purposes has also been reported in CHD patients [29]. The use of CAM in patients with CHD has been mapped in Norway, finding that 6.4% of patients with CHD had visited a CAM provider within the last 12 months [30]. Examining CAM use and the factors associated with CAM use among patients with CHD, and with non-modifiable risk factors for CHD, would contribute to the limited knowledge on CAM use in these groups.

The primary aim of this study was to examine the prevalence and predictors of CAM use in CHD patients, and in those without CHD but with non-modifiable risk for developing CHD, using PMT as a guiding conceptual framework. Based on current theory and evidence, we hypothesized that the set of PMT factors would be significant predictors of CAM use in the CHD family risk group and to a lesser extent in the CHD patient group.

## Method

The sixth Tromsø study is part of a single-centered population based health survey of adult inhabitants of the municipality of Tromsø in the northern part of Norway. It is a collaborative study in the interface between epidemiology and clinical medicine, including a main study that comprised two screening visits, two questionnaires and several follow-up studies [31]. The design includes repeated population health surveys to which total birth cohorts and random samples are invited.

To the sixth study, conducted in 2007–2008 the following subjects were invited: All participants who participated in the 2.nd visit of the fourth Tromsø study (1994–1995), a random sample of 10% of all inhabitants in the municipality of Tromsø aged 30–39 years, all inhabits in the municipality of Tromsø aged 40–42 years and 60–87 years, in addition to a random sample of 40% of all inhabitants in the municipality of Tromsø aged 43–59. A total of 12,982 people participated, with a response rate of 65.7%.

Individuals who attended the study by undergoing a health screening and answering the first questionnaire, received a second, more detailed questionnaire to complete and return at site of the health screening or later by mail. The questionnaires included questions about self-reported health, diseases suffered by the respondent or close family, food-, alcohol- and smoking habits, physical activity, education, general use of medication, and health services including CAM, not related to any specific condition or disease. The material used in this study is drawn from the two questionnaires described above. The population of Tromsø reflects the distribution of gender, and average income in Norway, although somewhat younger [32] and higher educated [33].

### Measures

**Fig. 1:**
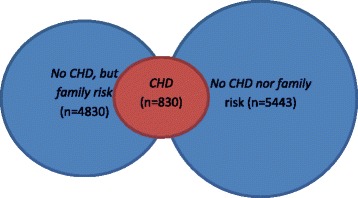
The participants divided in the studied groups

The three main groups describing the different CHD profiles were defined as follows (Fig. 1): *The CHD group*: People reporting to have or have had heart attack and/or angina pectoris regardless of family risk, *The no CHD but family risk group*: People *not* reporting to have or have had CHD, *but* who have parents, children or siblings with heart attack and/or angina pectoris and the *The no CHD nor family risk group*: People *not* reporting to have or have had CHD, nor parents, children or siblings with heart attack and/or angina pectoris.

The definition of a CAM user was based on three questions, one from the first questionnaire following the invitation to participate in the study (question 1), and two from the second questionnaire recieved on site of the health screening (question 2 and 3). Over all users of CAM consisted of informants who indicated YES for at least one of the following questions:

1. *Have you during the past year visited an alternative medical practitioner (homeopath, acupuncturist, foot zone therapist, herbal medicine practitioner, laying on of hands practitioner, healer, clairvoyant* etc.*)?*
2. *Have you in the last 12 months used herbal or natural medicine?*
3. *Have you in the last 12 months used meditation, yoga, qi gong or thai-chi as self-treatment?*

CAM was studied and described specifically for overall CAM use, as well as specifically for

1. Use of a CAM provider, 2. Over the counter (OTC) CAM products, and 3. CAM self-care approaches.

We first compared the patterns of CAM use between participants with CHD, and participants at risk of developing CHD, with participants with no CHD nor risk. Based on PMT, we hypothesized that those at risk for CHD would use more CAM than the other two groups as a means to reduce their risk for developing CHD. Risk for CHD was in this study conceptualized by having a family history of CHD (i.e., parents, children, or siblings with CHD).

### PMT variables

Health vulnerability was assessed from the following two questions about self-rated health and future health beliefs: *How do you in general consider your own health to be? (Very bad, Bad, Neither good nor bad, Good, Excellent)* and *I have a positive view of my future health, (1. disagree completely, 7. agree completely)*

Response efficacy was assessed from two measures, the first based on the questionnaire sentence *I can prevent serious diseases by living healthy, (1. disagree completely, 7. agree completely)*. The second measure was assessed with a health behavior index. Although PMT is often used to predict health behaviors [19], we considered that engaging in health-promoting behaviors would be reflective of efficacy for taking behavioral steps to reduce perceived risk for developing CHD, and thus increase the chances that CAM use would also be used to further reduce risk, especially among those with non-modifiable risk factors for CHD. The health behavior index was created from the mean score of responses to questions about the frequency of physical activity, eating fruit, vegetables and berries, using omega 3 capsules and cod liver oil, and less use of alcohol and beverages with sugar. Higher scores on the health behavior index indicated a more frequent practice of healthy behaviors.

We then examined the PMT predictors of CAM use. The health vulnerability from the threat appraisal process of PMT was assessed as current self-rated health and expectations for future health. Current and expected future health rated as poor were regarded as an indication of perceived vulnerability for CHD. Response efficacy from the coping appraisal process of PMT was assessed as endorsing preventive health beliefs (i.e., that healthy living can reduce the risk of disease), and engaging in more frequent healthy behaviours.

We defined three education response categories from the original five: low (primary and part of secondary school), middle (high school) and high (college or university) education. The income variable referred to the household’s total gross income last year. Eight original response categories were merged into low income (< NOK 301,000 (€ 34,000)), middle income (NOK 301,000–700,000 (€34,000–80,000)), and high income (> NOK 700,000 (€ 80,000)).

### Statistical analysis

Between-group differences were analyzed using chi-square tests for binary data analyzing one variable at the time. Bivariate correlations among the PMT variables were conducted with list wise deletion, stratified by each of the three risk groups, to provide essential information for understanding how each of these variables were inter-related prior to assessing their roles in the multivariate models. Three logistic regressions, one for each risk group, were conducted to identify the variables associated with CAM use from the set of PMT variables, while controlling for age, sex, marital status, and income. All analyses were conducted using SPSS for Windows (version 22.0, SPSS, Inc., Chicago, IL). The significance level was set to *p* < 0.05.

## Results

### Basic characteristics of the participants

**Fig. 2:**
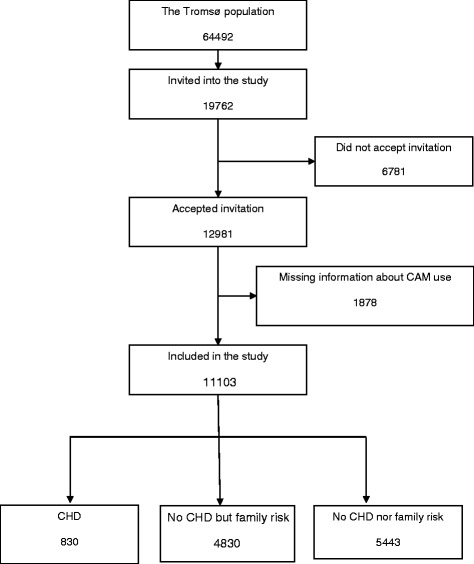
Flow chart of the included participants

The studied population consisted of 5876 (52.9%) women and 5227 (47.1%) men. Coronary heart disease was reported in 7.47% (*n* = 830) of the participants, 43.5% (*n* = 4830) had a family risk of CHD but no CHD themselves, while 49% (5443) were neither at risk nor diagnosed with CHD (Fig. 2). The CHD group consisted of more men than women. The family risk group consisted of more women than men while the no CHD nor risk group were gender balanced. Health was poorer and education lower in the CHD group compared to the *no CHD but family risk* and *no risk nor CHD* groups (Table 1).

**Table 1:** Basic characteristics of the participants

	CHD (*n* = 830^*^)	Family risk (*n* = 4830*)	No CHD nor Risk (*n* = 5443*)	*p*-value
	% (*n*)	% (*n*)	% (*n*)	
Mean age	68.46	57.07	54.67	< 0.001^^^
Gender				< 0.001^*^
Men	65.5 (552)	41.9 (2022)	48.7 (2653)	
Women	33.5 (278)	58.1 (2808)	51.3 (2790)	
Marital status				< 0.001^*^
Single	8.1 (67)	17.2 (829)	20.8 (1134)	
Married	62.9 (522)	60.4 (2917)	58.4 (3179)	
Separated/divorced	14.1 (117)	15.1 (728)	14.1 (766)	
Widowed	14.9 (124)	7.4 (356)	6.7 (363)	
Living with a spouse/partner				0.002^*^
Yes	72.0 (581)	75.1 (3541)	77.1 (4070)	
No	28.0 (226)	24.9 (1172)	22.9 (1209)	
Self-reported health			0.000^a^	
Bad	12.2 (100)	4.8 (231)	4.4 (240)	
Neither good nor bad	47.1 (385)	27.6 (1324)	25.0 (1352)	
Good	40.7 (333)	67.6 (3246)	70.6 (3818)	
Education				< 0.001^*^
Low	44.5 (362)	26.5 (1267)	24.1 (1298)	
Middle	32.5 (264)	35.2 (1686)	32.1 (1730)	
High	23.0 (187)	38.3 (1831)	43.8 (2360)	
Income				< 0.001^*^
Low	45.3 (340)	22.7 (1034)	19.2 (980)	
Middle	45.8 (344)	50.9 (2315)	50.7 (2586)	
High	8.9 (67)	26.4 (1201)	30.1 (1533)	

^*^Pearson’s Chi-Square test

^^^One-way ANOVA test

### Prevalence of CAM use

Over all CAM use was most commonly seen in the group *No CHD but family risk* (35.8%, *n* = 1730) followed by the *No CHD nor risk* group (32.1%, *n* = 1749) and *CHD* group (30.2%, *n* = 251, *p* < 0.001). This was also the case for CAM providers and OTC CAM products when the sub categories were analyzed separately. CAM self-care approaches was, however mostly used in the group with *No CHD nor family risk* (Table 2).

**Table 2:** CAM Use Within the Last 12 Months

	CHD (*N* = 830)	No CHD but family risk (*N* = 4830)	No CHD nor family risk (*N* = 5443)	*P*-value^*^
	% (*n*)	% (*n*)	% (*n*)	
CAM provider	9.4 (88)	12.8 (663)	11.6 (672)	0.007
OTC CAM products	20.8 (189)	25.4 (1273)	21.3 (1217)	<0.001
CAM self-care approaches	1.6 (15)	5.2 (261)	5.5 (314)	<0.001
Over all CAM use	30.2 (251)	35.8 (1730)	32.1 (1749)	<0.001

^*^Pearson’s Chi-square test

### Associations of PMT variables among CAM users

In all the three CHD groups self-rated health was correlated with *positive perceptions of future health*, *a belief that serious illness can be prevented by living healthy*, but not with *self-reported health behaviors (the health behavior index).* Preventive health beliefs were significantly correlated with beliefs about future health (Table 3).

**Table 3:** Means, Standard Deviations (SD), and Bivariate Correlations Among the Health-Related Beliefs and Behavior Variables for Each of the Coronary Heart Disease (CHD) Groups

Variable	1	2	3	4	Mean	SD
A. CHD (*N* = 244)						
1. Self-rated health	---				69.45	16.72
2. Health behavior index	.092	---			14.14	3.28
3. Preventive health beliefs	.253**	.047	---		5.89	1.34
4. Future health beliefs	.595**	.059	.339**	---	4.56	1.43
B. No CHD but family risk (*N* = 1875)						
1. Self-rated health	---				77.94	15.76
2. Health behavior index	.012	---			14.26	3.23
3. Preventive health beliefs	.186**	.093**	---		6.02	1.14
4. Future health beliefs	.606**	.051	.420**	---	5.07	1.37
C. No CHD nor family risk (*N* = 2267)						
1. Self-rated health	---				78.87	15.50
2. Health behavior index	.016	---			13.83	3.32
3. Preventive health beliefs	.210**	.053*	---		6.07	1.14
4. Future health beliefs	.574**	.054*	.415**	---	5.22	1.36

*Note:* *p < .01, **p < .001. *N’*s adjusted by list wise deletion

#### CHD group

In those already diagnosed with CHD, current health behaviors were not significantly associated with health beliefs.

#### No CHD but family risk group

In this group, current practice of health behaviors were significantly associated with preventive health beliefs, but not with a positive belief about future health.

#### No CHD nor risk group

The health behavior index was significantly and modestly associated with both health beliefs (preventive and future health) in this group only.

### Predictors of CAM use as a function of CHD Group

The results of the logistic regressions predicting CAM use for each CHD group, revealed a pattern of associations that was generally consistent with PMT (Table 4). When adjusted for socio-demographic variables, all four of the PMT factors - *self-rated health (OR 0.78, CI 0.69–0.88), expectations for future health (OR 0.91, CI 0.85–0.98), preventive health beliefs (OR 1.09, CI 1.01–1.17), and the health behavior index* (OR 1.12, CI 1.09–1.14) were significant predictors of CAM use for those with *no CHD but family risk*. In the *CHD* group, only the *health behavior index* (OR 1.12, CI 1.05–1.20) and *self-rated health* (OR 0.72, CI 0.52–0.99) were significantly associated with CAM use. In the *no CHD nor family risk* group, *self-rated health (OR 0.88, CI 0.72–0.90), the health behavior index (OR 1.10, CI 1.07–1.12), and future health beliefs (OR 0.90, CI 0.85–0.96)* were the only PMT variables significantly associated with CAM use. *Preventive health beliefs* were not a significant predictor.

**Table 4:** Adjusted odds ratios (ORs) and 95% confidence intervals (95% CI) of factors independently associated with the use of CAM, stratified by no coronary heart disease (CHD) but family risk group

	CHD (*n* = 466)^*^			No CHD but family risk (*n* = 3360)^*^			No CHD nor family risk (*n* = 3838)^*^		
	OR	95% CI	*p*-value	OR	95% CI	*p*-value	OR	95% CI	*p*-value
Age	0.98	0.96–1.01	0.235	0.99	0.98–0.99	0.013	1.00	0.99–1.00	0.430
Sex^a)^									
2.23	1.18	0.72–1.92	0.517	2.30	1.96–2.70	2.23	2.23	1.92–2.59	< 0.001
Marital status^a)^									
1.00	1.00	---	0.319	1.00	---	1.00	1.00	---	0.488
0.99	0.76	0.28–2.07	0.594	1.07	0.72–1.61	0.99	0.99	0.66–1.48	0.941
1.03	0.66	0.32–1.36	0.261	1.14	0.80–1.64	1.03	1.03	0.71–1.50	0.861
1.0	1.13	0.50–2.57	0.766	1.12	0.76–1.66	1.0	1.0	0.79–1.77	0.408
Income^a)^									
1.0	1.0	---	0.597	1.0	---	1.0	1.0	---	0.313
1.17	1.56	0.62–3.51	0.325	1.07	0.85–1.66	1.17	1.17	0.91–1.52	0.220
1.13	1.46	0.67–3.21	0.344	1.27	0.39–0.92	1.13	1.13	0.95–1.34	0.156
Self-rated health	0.72	0.52–0.99	0.044	0.78	0.69–0.88	0.000	0.80	0.72–0.90	< 0.001
Health behavior index	1.12	1.05–1.20	0.001	1.12	1.09–1.14	0.000	1.10	1.07–1.12	0.001
Preventive health beliefs	1.00	0.85–1.17	0.971	1.09	1.01–1.17	0.019	1.07	1.00–1.14	0.056
Future health beliefs	0.99	0.84–1.18	0.944	0.91	0.85–0.98	0.008	0.90	0.85–0.96	0.002

^*^ *Note: N’*s adjusted by list wise deletion. ^a)^Reference categories were *male*, *single* and *low income*

The socio-demographic predictors of CAM use also varied as a function of CHD risk groups. Being female was associated with CAM use in both the *No CHD nor family risk* (OR 1.92, CI 1.92–2.59) and the *No CHD but family risk* groups (OR 2.30, CI 1.96–2.70), but not the *CHD* group. Being younger was linked to CAM use in the *No CHD but family risk* group only (OR 0.99, CI 0.98–0.99).

## Discussion

Use of CAM was most commonly seen in people with no CHD themselves, but at risk of developing CHD. All four of the PMT factors - self-rated health, expectations for future health, preventive health beliefs, and the health behavior index – were associated with CAM use in the risk group. This provides suggestive evidences that people use CAM in response to a health threat, to prevent disease and to maintain health. The socio-demographic factors associated with CAM use were generally in line with those found for other chronic health conditions [34], with being female and younger linked to CAM use in some, but not all of the CHD risk groups. Consistent with PMT [17, 18], use of CAM was more common among people with *no CHD but family risk* than in people with *no CHD nor family risk* or already diagnosed with *CHD*. The full PMT set of variables was also a significant predictor of CAM use in the *No CHD but family risk* group.

### Prevalence of CAM use in CHD

Our findings of 30.2% CAM use in patients already diagnosed with CHD is in accordance with a resent review finding that 4–61% of the cardiac patients across 27 studies reported to use CAM [27]. The findings of 9.4% use of a CAM provider is however somewhat higher than what was found in the fifth Tromsø study conducted 6 years earlier [30]. The main reason for this increase might be a general increase in the use of CAM and a pre-prepared list of CAM providers presented in the questionnaire that might have improved the recall and clarified what to consider as CAM [35]. The higher use of a CAM provider in the *No CHD nor family risk* group is interesting and partly in accodance with what was found in the fifth Tromsø study where 6.5% in the *CHD* group and 9.5% in the *No cancer nor CHD* group reported to have seen a CAM provider. The reason for higher use of CAM in the *No CHD nor family risk* group than in the *CHD* group might therefore partly be due to the gender- and age differences found in these groups, as older males are known to be less frequent users of CAM [35]. The lower use of CAM in the *CHD* group than in the *No CHD but family risk* group, might in addition be due to the fact that patients already diagnosed with CHD are taken care of within the conventional health services to a greater extent than people with a family risk only.

### PMT correlates of CAM use across CHD risk groups

Framed from a PMT perspective, our findings indicate that people with a family history that puts them at risk for developing CHD, use CAM as a health-promoting behavior to cope with, and minimize this perceived risk. In this respect, our findings extend previous research indicating that CAM users are proactive in their approach to health [34, 36, 37], and that CAM is used alongside other important health-promoting and preventive behaviors [8]. The high use of CAM in the group with *no CHD but family risk* is also in accordance with findings showing that one of the main reasons for CAM use is disease prevention [38], and that CAM use is associated with reducing risk factors such as being a former smoker [8]. Our findings also highlight that CAM use may be motivated by a protective response to perceptions of risk for developing specific diseases, such as CHD, and not just as a means of general disease prevention. Indeed, the belief that by living healthy one can avoid disease, was a significant predictor of CAM use only in those with *no CHD but family risk*, and therefore perceived risk of developing CHD, but not among those with *no CHD nor family risk*.

### Strengths and limitations

The main strength of this study is the large population-based sample and the rather high response rate in addition to the rich information about health-related issues. Despite a high response rate, our sample may not be entirely representative of the general population, as women, married people, healthy people and higher socio-economic groups are more likely to participate in population surveys. In this particular study the participants were older and the proportions of married people and women were higher than for non-attendees [39].

Another limitation is that all information is self-reported and may therefore be inaccurate due to recall bias and individual definitions of CAM and heart attack/angina pectoris. Further, the construction of the categories including only angina pectoris and heart attack in the CHD group has some limitations when comparing CAM use in this group with other studies. As well, the data was collected at a single time point, making it difficult to draw any causal conclusions about the relationships between the PMT variables and CAM use. Nonetheless, our suggestion that the variables predicted CAM use in the regressions rather than vice versa, was informed by established theory, PMT, which suggests that threat and coping appraisals play a role in the behavioral responses to perceived risk for illness [19]. Finally, the risk groups were based on family history of CHD rather than on any objectively measured physical risk factors, such as hypertension, or high cholesterol, and therefore the groups may not have been accurate with respect to *actual* risk for CHD. However, we argue that it is the *perceived* risk for CHD that is important in terms of people’s choice to use CAM, and consistent with PMT [13, 14]. Feeling vulnerable because of a family history of CHD may be enough to motivate people to use CAM as a means to help reduce this perceived threat.

## Conclusions

This is one of few studies mapping CAM use in people with coronary heart disease in Norway, and to our knowledge, the first to apply PMT to understand the health-related beliefs and behaviors associated with CAM use in people at risk of developing CHD. The study might therefore be a door opener to the field. Future research should examine the extent to which CAM is used to prevent specific diseases in response to perceived risks, and as a primary prevention strategy among individuals with known risk factors like a family history of disease, diagnoses of specific disease precursors for CHD, for example obesity, high cholesterol, and hypertension.

## Abbreviations

CAM: Complementary and alternative Medicine
CHD: Coronary heart disease
PMT: Protection Motivation Theory
